# Nitidine chloride possesses anticancer property in lung cancer cells through activating Hippo signaling pathway

**DOI:** 10.1038/s41420-020-00326-7

**Published:** 2020-09-19

**Authors:** Jing Zhang, Linhui Wu, Chaoqun Lian, Shuo Lian, Shimeng Bao, Jisheng Zhang, Peter Wang, Jia Ma, Yuyun Li

**Affiliations:** 1 Department of Genetics, School of Life Sciences, Bengbu Medical College, Anhui, 233030 China; 2 Bengbu Medical College Key Laboratory of Cancer Research and Clinical Laboratory Diagnosis, Bengbu Medical College, Anhui, 233030 China; 3 Department of Biochemistry and Molecular Biology, School of Laboratory Medicine, Bengbu Medical College, Anhui, 233030 China; 4 School of Clinical Medicine, Bengbu Medical College, Anhui, 233030 China; 5 School of Pharmacy, Bengbu Medical College, Anhui, 233030 China; 6 School of Life Sciences, Bengbu Medical College, Anhui, 233030 China; 7 Department of Laboratory Medicine, School of Laboratory Medicine, Bengbu Medical College, Bengbu, Anhui 233030 China

**Keywords:** Non-small-cell lung cancer, Preclinical research

## Abstract

Nitidine chloride (NC) has significant anti-tumor properties; however, the precise mechanism related to NC still needs further investigation. This study intends to investigate the anti-tumor functions and the feasible molecular basis of NC in NSCLC cells. Therefore, we determined the mechanism of NC-mediated anti-tumor function through various methods. Cell proliferation ability and migration and invasion were detected by CCK-8, colony formation assay and Transwell assay, respectively. Furthermore, flow cytometry was used to detect apoptosis, cell cycle and ROS. Moreover, protein expression level was measured by western blot. Our results showed that NC can inhibit the growth, motility of NSCLC cells, induce apoptosis and arrest cell cycle. Meanwhile, NC increased the level of ROS in NSCLC cells. Moreover, western blot data showed that NC suppressed the expression of Lats1, Mob1, and YAP, and enhanced the expression of p-Lats1, p-Mob1, p-YAP1 (ser127). Overall, our research reveals that NC exerts anticancer activity by activating and modulating the Hippo signaling pathway.

## Introduction

Non-small cell lung cancer (NSCLC) is a malignant tumor that poses a serious threat to human health^1^. The treatment of NSCLC patients is mainly based on postoperative chemotherapy combined with radiotherapy^2^, but the side effects of radiotherapy and chemotherapy are very conspicuous, and patients will eventually develop drug resistance leading to treatment failure^3^. Thus, it is necessary to find key target molecules and new therapeutic strategies to inhibit the progress of NSCLC.

In recent years, the newly discovered Hippo signaling pathway has gradually received extensive attention. Studies have shown that its abnormalities are often involved in the occurrence and development of human tumors. A series of conserved protein kinases constitute the core components of Hippo signaling pathway. In mammals, Hippo pathway includes mammalian Sterile 20-like kinase 1 and 2 (MST1/2), large tumor suppressor kinase 1 and 2 (LATS1/2), and Mps one binder protein 1 (MOB1), Yes-associated protein (YAP), and transcriptional coactivator with PDZ-binding motif (TAZ)^4,5^. When the growth inhibitory signal is transmitted to the cell membrane, the Hippo signal is activated, then MST1/2 and WW45 phosphorylate and activate the downstream LATS1/2 kinase and adaptor protein MOB1, and then phosphorylate the downstream transcriptional regulator YAP/TAZ. As an oncogene, YAP/TAZ promotes excessive cell proliferation and counteracts apoptosis^6^. Phosphorylated YAP/TAZ binds to 14-3-3 in the cytoplasm and gradually degrades and ubiquitinates, thus failing to enter nuclear transcription to initiate target gene expression, and ultimately inhibits organ size, tumor cell proliferation, and metastasis^5,7^. The Hippo pathway is very conservative during evolution, and it is crucial in regulating cell proliferation, differentiation, tissue regeneration, organ size, as well as cancer development^8–10^. In a variety of human tumors, Hippo signaling pathway is downregulated, suggesting that it is closely related to tumorigenesis^11,12^. Evidence also indicates that Hippo signaling pathway plays a key role in the development of NSCLC^4^. Consequently, it is very important to develop new anti-cancer therapies through targeting Hippo pathway.

Nitidine chloride (NC) is a pure natural alkaloid with biological activity^13^. Previous studies revealed that NC has multiple pharmacological functions, such as anti-fungal, anti-inflammatory, and anti-oxidant properties^14,15^. Recently, several studies revealed tumor-suppressive functions of NC in various types of human malignant cancers^16–19^. As a natural anti-cancer drug, NC has received extensive attention. We previously demonstrated that NC had an inhibitory effect on prostate cancer cells and osteosarcoma cells^20,21^. However, the role of NC in human lung cancer cells and its potential molecular mechanism need to be further elucidated. In this study, we sought to investigate the role of NC in inhibiting the biological function of NSCLC cells. Moreover, whether its molecular mechanism is related to the Hippo pathway was further explored.

## Results

### NC inhibited cell viability and colony formation

**Fig. 1:**
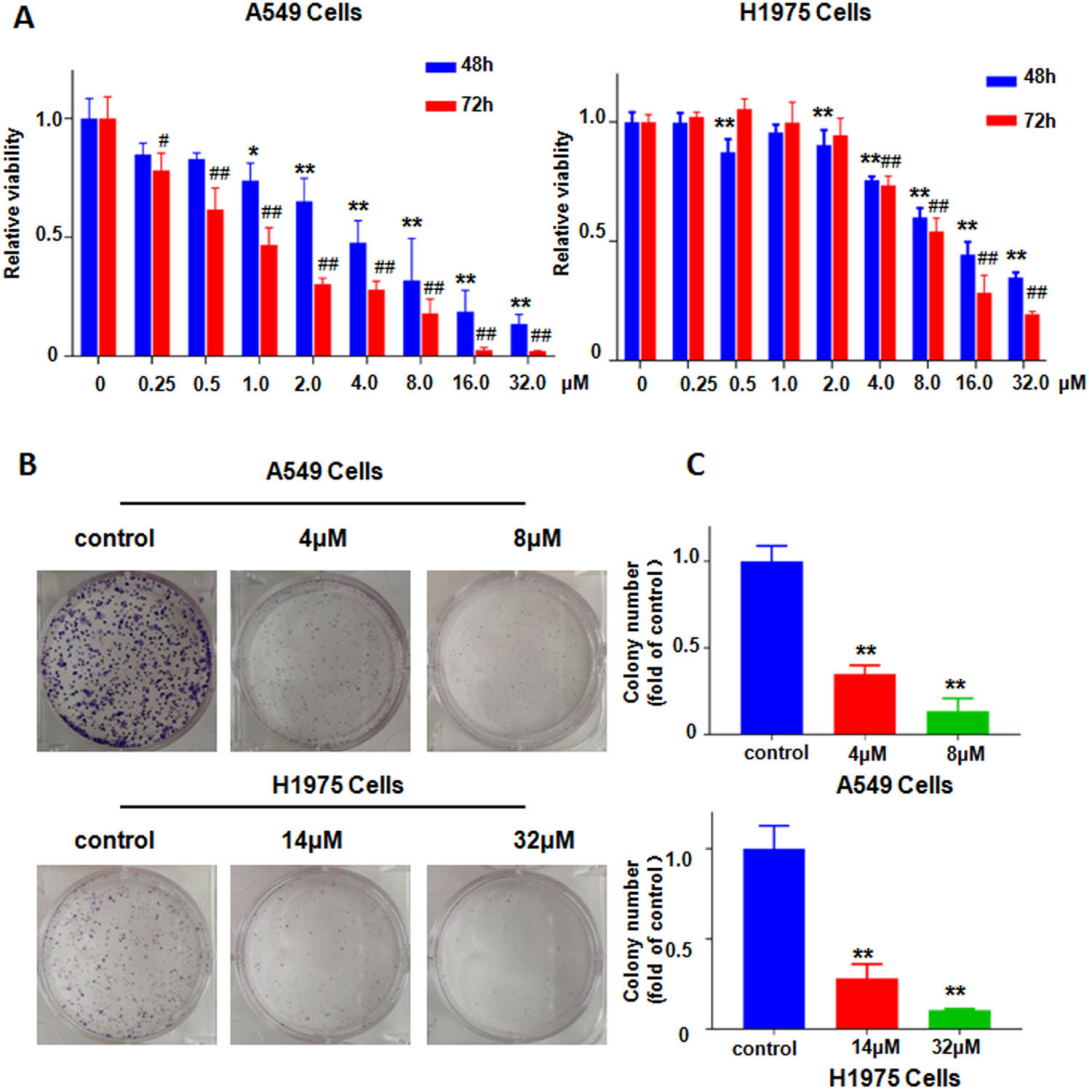
NC restrained viability of NSCLC cells. **a** The viability of A549 and H1975 cells was detected by CCK-8 assay after treatment with various concentrations of NC for 48 h and 72 h. *P < 0.05, **P < 0.01 vs. control (48 h); ^#^*P* < 0.05, ^##^*P* < 0.01 vs. control (72 h). **b** Colony formation assay was performed in A549 and H1975 cells treated with NC at the indicated concentration for 48 h. **c** Quantification of the colony number in (**b**). **P < 0.01 vs. control, the same in the following figure legends.

CCK8 assays and colony formation assays were performed in NSCLC cells. In CCK8 assays, A549 and H1975 cells were incubated in different concentrations of NC (0.25, 0.5, 1, 2, 4, 8, 16, 32 μM) for 48 and 72 h, respectively. The results showed that NC significantly inhibited the viability of NSCLC cells compared to the control group with 0.1% DMSO alone, and this effect was more pronounced with increasing doses and time (Fig. 1a). Almost 50% cell viability in A549 and H1975 cells was suppressed by 4 μM and 14 μM NC after 48 h treatment, respectively. NC induced about 70% inhibition of cell viability in A549 cells and H1975 cells treated with 8 μM and 32 μM for 48 h, respectively. Therefore, we chose 4 μM and 8 μM NC for A549 cells and 14 μM and 32 μM NC for H1975 cells to conduct subsequent experiments. Next, the effect of NC treatment on the number of cell-forming clones was further examined by colony formation assay. The results showed that as the concentration increased, NC significantly reduced the number of colonies in A549 and H1975 cells (Fig. 1b, c). Altogether, NC effectively suppressed cell viability in NSCLC cells.

### NC inhibited motility of NSCLC cells

**Fig. 2:**
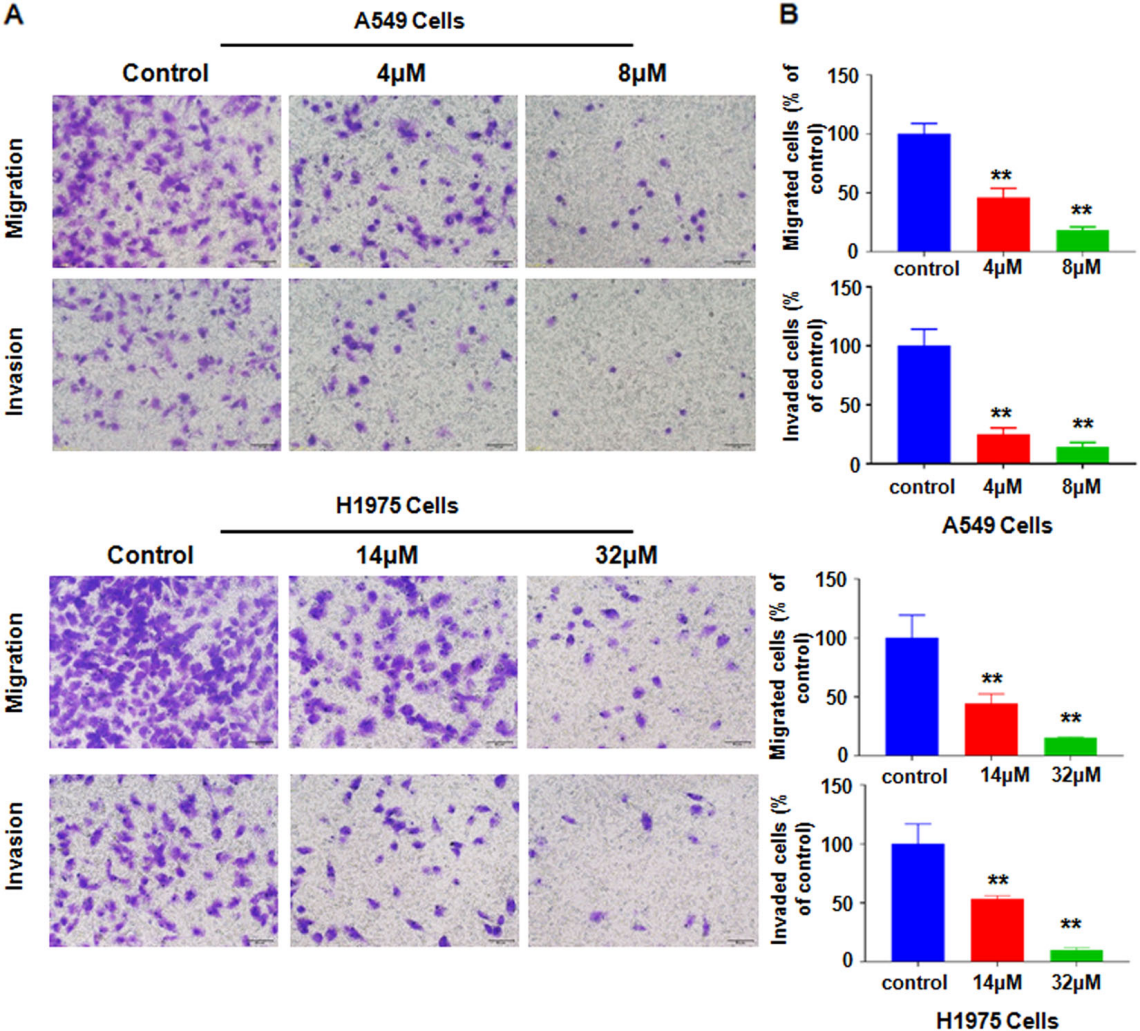
NC inhibited motility of NSCLC cells. **a** NSCLC cells were treated with different doses of NC for 24 h. Transwell chamber assay was used to detect the migratory and invasive ability of NSCLC cells. Take photos of cells that have migrated or invaded to the lower surface in Transwell analysis (×200). **b** Quantitative analysis was illustrated for the cells of migration and invasion. **P < 0.01 vs. control.

Transwell assay further dissects the effects of NC on motility ofA549 and H1975 cells in vitro. As shown in Fig. 2a, b, the results from transwell showed that as the NC concentrations were increased, A549 or H1975 cells that migrated and invaded into the lower chamber became less. Due to that NC did not inhibit cell viability in A549 and H1975 cells at 24 h when these cells were cultured without FBS (data not shown), our results revealed that NC inhibited motility of NSCLC cells.

### NC induced cell apoptosis

**Fig. 3:**
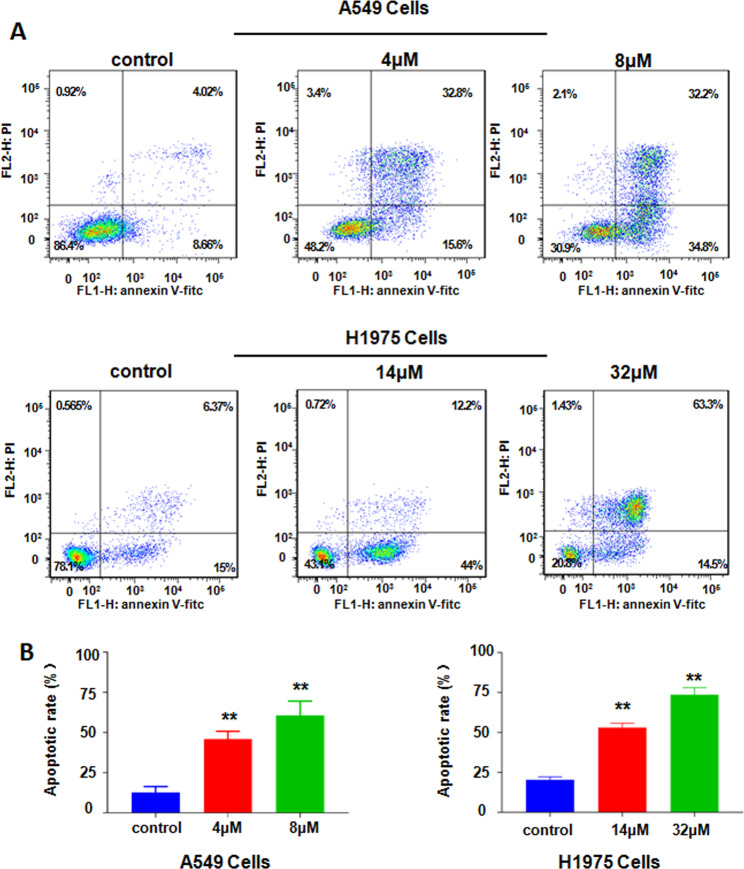
NC induced apoptosis in NSCLC cells. **a** Flow cytometry analysis of cell apoptosis in A549 and H1975 cells was determined after treating with different doses of NC for 48 h. **b** Quantitative analysis of percentages of apoptotic cells in the A549 and H1975 cells. **P < 0.01 vs. control.

Annexin V-FITC/PI double fluorescence staining and flow cytometry were used to detect apoptosis in NSCLC cells treated with NC for 48 h. The results showed that NC treatment enhanced cell apoptosis. The apoptosis rates of A549 cells were increased from 12.58 ± 2.20% of the control group to 45.97 ± 2.75% and 60.73 ± 5.05% following exposure to NC (4 and 8 µM), respectively (Fig. 3a, b). And treatment with NC (14 and 32 µM) in H1975 cells caused 53.07 ± 1.59% and 73.6 ± 2.55% apoptosis rate (Fig. 3a, b). The results dissected that NC significantly stimulated NSCLC cell apoptosis.

### NC caused cell cycle arrest

**Fig. 4:**
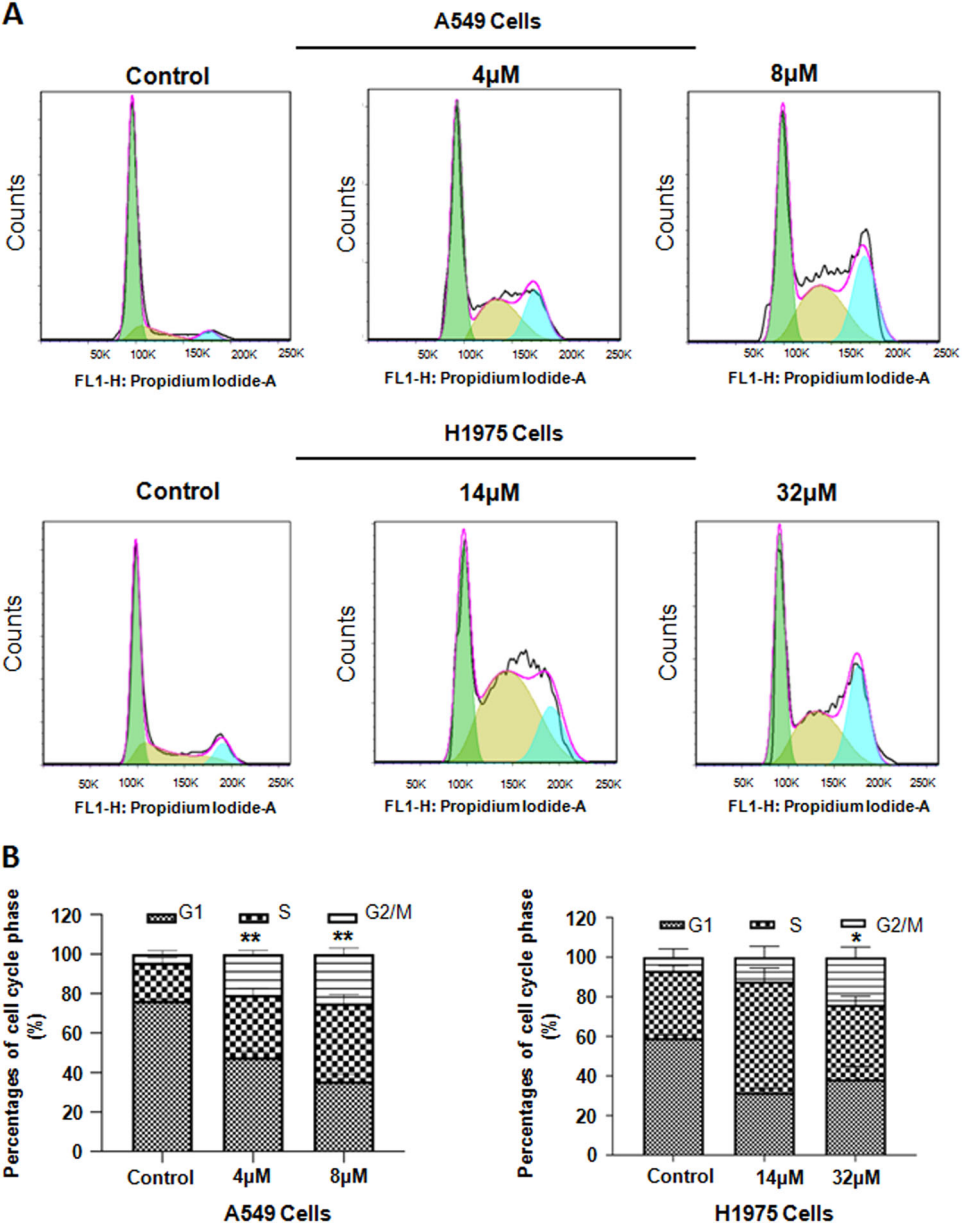
NC caused cell cycle arrest of NSCLC cells. **a** Cell cycle in A549 and H1975 cells was detected by PI staining after treating with different doses of NC for 48 h by flow cytometry. **b** The percentage of each cell cycle phase of A549 and H1975 cells was illustrated. *P < 0.05, **P < 0.01 vs. control.

Through the detection by flow cytometry, we could acquire the distribution of cell cycle at each phase. Compared to control, NSCLC cells treated with NC were arrested at the G2/M phase. As NC concentrations increased, the proportion of block at the G2/M phase gradually increased (Fig. 4). Compared with the control groups, the average proportion of G2/M phase for A549 cells at the concentration of 4 μM was increased, and the average proportion of G2/M phase for H1975 cells in at the concentration of 32 μM was increased (Fig. 4).

### NC increased ROS level

**Fig. 5:**
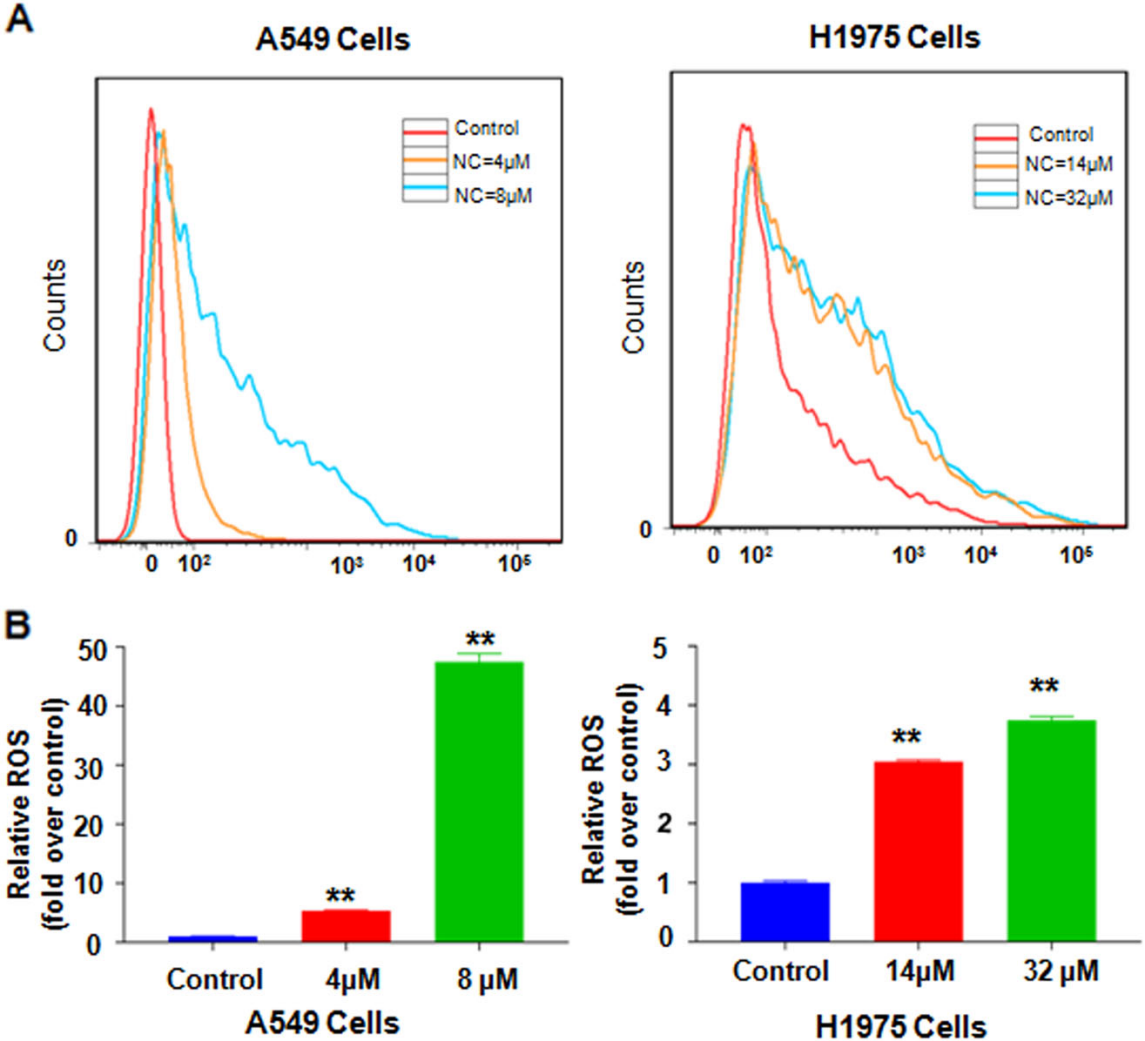
NC promoted ROS level in NSCLC cells. **a** ROS level in A549 and H1975 cells was detected by DCFH-DA staining after treating with different doses of NC for 48 h by flow cytometry. **b** The percentage of relative ROS compared with control in A549 and H1975 cells was illustrated. **P < 0.01 vs. control.

There were 10000 cells collected in each group. As shown in Fig. 5a, b, NC could significantly enhance the level of ROS species. Increased levels of intracellular ROS were concentration-dependent. Compared with H1975 cells, the response of A549 cells to NC was more sensitive. When treated with about 50% inhibiting concentration of NC, the level of ROS in A549 and H1975 cells was about 47.4 times higher and 3.74 times higher than that of the control, respectively (Fig. 5a, b).

### NC activated the Hippo signaling pathway

**Fig. 6:**
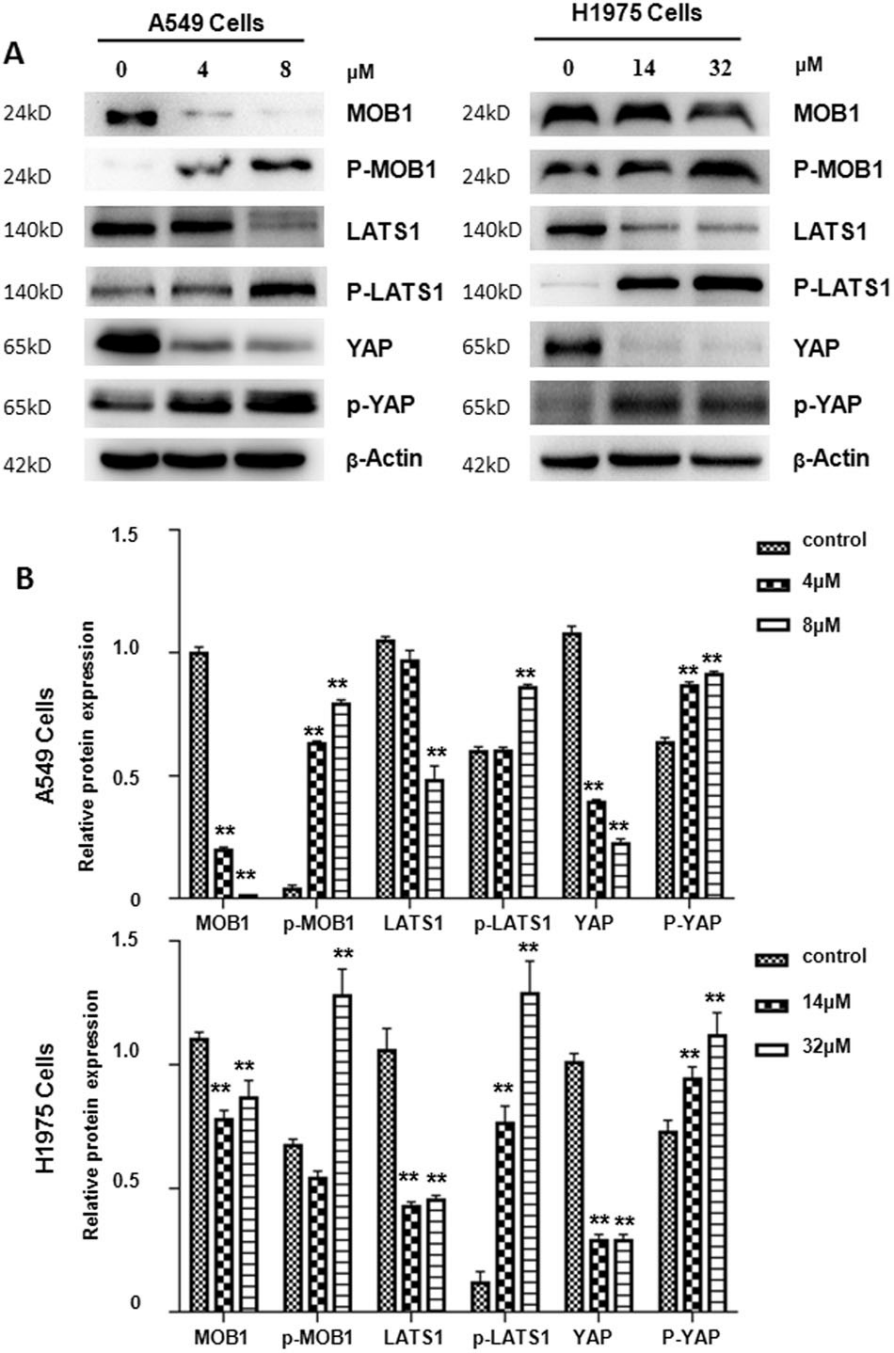
NC activated Hippo pathway of NSCLC cells. **a** Following different doses of NC treatment in A549 and H1975 cells for 48 h, the protein levels of MOB1, p-MOB1^Thr35^, LATS1, p-LATS1^Thr1079^, YAP, and p-YAP^Ser127^ were evaluated by immunoblot. **b** Statistical analysis on relative protein expression of Hippo pathway in A549 and H1975 cells. **P < 0.01 vs. control.

Previous evidence indicates that the Hippo pathway is associated with the occurrence and development of NSCLC. However, it was not clear whether NC affected NSCLC cells through Hippo pathway. To test this possibility, the expression of key proteins in the Hippo pathway was detected after 48 h treatment by NC. As depicted in Fig. 6a, b, the levels of MOB1, LATS1, and YAP were markedly reduced under NC treatment. Meanwhile, compared with control, NC treatment significantly enhanced the levels of p-MOB1, p-LATS1, p-YAP1 (ser127). These findings indicate NC could activate the Hippo pathway.

## Discussion

In recent years, research on Hippo pathways in tumor development has been deepened. It was reported that the Hippo signaling pathway is related to the progression of lung cancer. Studies show that YAP1 mutation leads to higher susceptibility to lung adenocarcinoma^22^. YAP is an important factor in the development and progression of lung cancer in mice^23^. Lau et al. found that the proliferation and metastasis of lung tumor cells were associated with YAP/TAZ, and YAP activation will affect the progress of lung tumors in vivo^24^. YAP inhibition reduced brain metastasis of lung adenocarcinoma cells in nude mice^25^. Interestingly, Hippo pathway is involved in the regulation of tumor immunogenicity^26^. The transcription of immune checkpoint PD-L1 was controlled by YAP in lung cancer^27,28^. Inhibition of PD-L1 can reduce the proliferation and wound healing of EGFR-TKI-resistant lung adenocarcinoma cells^28^. Therefore, these reports indicated that Hippo signaling pathway may provide new clues for lung cancer therapy.

Growing body of researches has been suggested that the increased ROS contributed to carcinogenesis, leading to activation of cell growth and survival. Furthermore, exaggerated generation of ROS in cancer cells trigged cell death, resulting in anti-tumor effects^29,30^. Some evidence report that oxidative stress regulates the Hippo pathway in cancer cells^31^. A recent study revealed that in malignant melanoma cells, ROS might be required for MST1 activation^32^. In gastric cancer cells, the production of ROS was significantly related to the anti-tumor activity induced by the Curcumin Analogue WZ35 and the down-regulation of YAP^33^. Inhibiting YAP can induce the accumulation of ROS, which helped to overcome the resistance to chemotherapy of hepatocellular carcinoma (HCC)^34^. Thus, our data demonstrated that NC could obviously increase ROS production in lung cancer cells, which leads to enhanced tumor suppression function of NC.

Natural products have gained extensive attention as potential anticancer drugs for more effective biological activities and low cytotoxicity. In previous studies, we revealed that NC can be used as a YAP inhibitor to treat prostate cancer cells^20^. In the present study, our data demonstrated NC inhibited cell viability, motility, caused cell cycle arrest, and promoted apoptosis and ROS level in NSCLC cells. We further explored the potential mechanism which NC affected NSCLC cells. Our findings indicated that NC could activate the Hippo pathway. All these data indicated that the Hippo pathway participates in the process of NC treatment in NSCLC cells.

## Materials and methods

### Cell culture and NC treatment

A549 and H1975 were provided by Chinese Academy of Science Cell Bank (Shanghai, China). RPMI-1640 medium, supplemented with 10% FBS and 1% penicillin–streptomycin mixture was used to culture cell lines. The cells were incubated in tissue culture dishes at 37 °C in 5% CO_2_ atmosphere. Nitidine chloride (NC, CAS no. 13063-04-2) was provided by Tauto Biotech Co. Ltd. (Shanghai, China). In order to guarantee the final concentration of dimethyl sulfoxide (DMSO) was 0.1%, NC was firstly dissolved in DMSO as 25 mM stocks, respectively, and stored at −20 °C. And then diluting it with RPMI-1640 medium to different concentrations of NC, and 0.1% DMSO was maintained in all samples.

### Reagents and antibodies

Reactive oxygen species (ROS) Assay Kit and Cell Counting Kit-8 (CCK-8) assay were provided by Beyotime Institute of Biotechnology (Shanghai, China). Matrigel and FITC Annexin V Apoptosis Detection Kit I (#556547) were provided by BD Biosciences (Franklin Lakes, NJ, USA). The secondary anti-mouse and anti-rabbit antibodies were provided by Cell Signaling Technology (Danvers, MA, USA). Transwell plates were obtained from Corning Incorporated (Corning, NY, USA).

### CCK-8 assay

Cell viability was detected by the CCK-8 assay. A549 or H1975 cells were seeded in a plate. After the cells were attached, each well was treated with NC or DMSO (vehicle control) and incubated for 48 and 72 h at specified concentrations. Each well was treated with 15 µl CCK-8 solution and incubated for 2 h. Measure absorption at 450 nm with a multi-functional microplate reader (BioTek, cytation3, USA).

### Colony formation assay

For each well, 1.5 × 10^3^ cell were seeded in 6-well-plate and incubated overnight, NC and DMSO were added to each well for 7 days in a humidified 5% CO_2_ atmosphere at 37 °C. Before the cells were fixed with 4% paraformaldehyde for 20 min, PBS was selected to wash cells for twice, and added crystal violet solution for staining and rinsed under running water. The visible colonies were counted by Imagej software.

### Transwell assay

In order to detect the capacity of NSCLC cells to invade and migrate, cells are planted in the transwell chamber according to the manufacturer protocol. Compared with migration assay, invasion assay is different in that the polycarbonate membrane is coated with 60 μl of 1 mg/ml matrigel for 2 h. The cells treated with NC or DMSO were harvested and suspended in RPMI-1640 medium without serum. Then, 4 × 10^4^ cells were transplanted into the upper inserts, and 700 µl completed RPMI-1640 medium was injected into lower chamber. After 24 h, cotton swabs were selected to remove non-migration or non-invasive cells. The cells which reached the lower surface through coated membranes were fixed by 4% PAF and stained by Crystal Violet solution. The invaded cells were photographed with a microscope, and counted at least six randomly-selected images to represent the relative migration and invasion.

### Cell apoptosis assay

A549 or H1975 cells were seeded in 6-well plates and treated with NC or DMSO for 48 h after the cells adhered. Cells were digested by trypsin without ethylenediaminetetaacetic acid (EDTA) and washed with pre-cooled PBS. The collected cells were suspended in 100 μL binding buffer (1×), then incubated with 5 μL FITC Annexin V and 5 μL PI for 15 min in the dark (25 °C), and then added 400 μL binding buffer (1×). Finally, the stained cells were measured within 1 h of staining by a FACSVerse flow cytometer (BD Biosciences, USA), then the measurement results of assay was analyzed by FlowJo software (version7.6.1; Treestar, USA).

### Cell cycle analysis assay

For determining the effect of NC on cell cycle, A549 or H1975 cells were cultivated in a 6-well-plate, used serum-free RPMI-1640 medium to grow to 70% confluency. Removed the serum-free medium and treated the cells with NC, respectively. After 48 h at 37 °C, the cells were digested by trypsin without EDTA, collected by centrifugation at 2000 rpm for 5 min, and then were fixed in cold ethanol for 24 h at 4 °C. The cells were washed with pre-chilled PBS and incubated with a propidium iodide solution containing RNaseA (0.1 g/mL) for 30 min in the dark at 37 °C. After cells were filtered through a nylon mesh (45-μm well), the distribution of cell cycle was processed by using flow cytometry. Finally, the measurement data were analyzed by Flowjo software. The difference among multiple groups was analyzed by one-way analysis of variance (ANOVA).

### ROS level assay

ROS in cells oxidized DCFH to generate fluorescent DCF which was then detected. Firstly, NSCLC cells were cultivated in a 6-well plate, and tests were carried out at different concentrations of NC in different groups. The cells in different sample-plates were collected respectively by trypsin digestion and centrifugation, incubated and stained with DCFH-DA for 30 min at 37 °C. Then, the samples were filtered through a nylon mesh (45-μm well) before it was analyzed by flow cytometry.

### Immunoblotting

A549 or H1975 cells treated with NC or DMSO were harvested and lysed to extract total protein from the cells in a cell lysis buffer supplemented with protease inhibitors. Then quantified by BCA protein assay, isocratic protein (60–80 μg) from cell lysate was separated by 10% SDS-PAGE and transferred to Immobilon®-P Transfer Membrane. The membrane was incubated with indicated primary antibody. The primary antibodies were obtained from Cell Signaling Technology Company, including p-YAP(Ser127, D9W2I, #13008, 1:1000), MOB1 (E1N9D, #13730, 1:1000), p-MOB1 (Thr35, D2F10, #8699, 1:1000), LATS1(C66B5, #3477, 1:1000), p-LATS1 (Thr1079, D57D3, #8654, 1:1000), YAP (#4912, 1:1000). The anti-actin (A1978, 1:5000) antibody was bought from Sigma-Aldrich. Tris buffered saline, containing Tween 20 (TBST), was selected to wash the membrane and then the membrane was applied to the secondary antibody was incubated for 2 h (25 °C). Finally, the chemiluminescent HRP substrate (Millipore Corpration, Billerica, MA, USA) was used and the immunoreactive bands were visualized by the Tanon5200 Detection System (Shanghai, China). ImageJ software was selected to analyze the protein expression intensity.

### Statistical analysis

All data were showed as mean ± standard deviation (SD). All data are representative of three independent experiments. All statistical analyses were performed by GraphPad Prism 6.0 software, and the data therein were expressed as mean ± standard deviation (SD). Comparison of multiple groups was checked by ANOVA. *P* < 0.05 and *P* < 0.01 indicate that the difference is statistically significant.
